# Neuromyelitis optica with brain stem involvement in a middle-aged Ethiopian woman: a case report and review of literature

**DOI:** 10.1186/s13256-021-03019-6

**Published:** 2021-10-01

**Authors:** Rodas Asrat Kassu, Hailu Abera Mulatu, Sisay Gizaw, Henok Fisseha, Amir Musema, Ayube Keder, Semere Negash, Fithanegest Tefera, Adugna Lissanwerk, Lemlem Tamrat

**Affiliations:** 1 Department of Neuro-surgery, St. Paul’s Hospital Millennium Medical College, Addis Ababa, Ethiopia; 2 Department of Internal Medicine, St. Paul’s Hospital Millennium Medical College, Addis Ababa, Ethiopia; 3 Department of Gynecology, St. Paul’s Hospital Millennium Medical College, Addis Ababa, Ethiopia; 4 Department of Radiology, St. Paul’s Hospital Millennium Medical College, Addis Ababa, Ethiopia; 5 Department of Ophthalmology, St. Paul’s Hospital Millennium Medical College, Addis Ababa, Ethiopia

**Keywords:** Ethiopia, Neuromyelitis optica, Optic neuritis, Transverse myelitis, Oculomotor, Brainstem, Case report

## Abstract

**Introduction:**

Neuromyelitis optica is a demyelinating disease of the central nervous system that predominantly affects the optic nerves and spinal cord. In neuromyelitis optica, white blood cells and antibodies primarily attack the optic nerves and the spinal cord, but may also attack the brain. Brainstem manifestation has been described recently. So far, neuromyelitis optica is very rare in Ethiopia and there were only two case reports, but this is the first case report of neuromyelitis optica with brainstem involvement.

**Case presentation:**

A 47-year-old Addis Ababa woman presented to Saint Paul’s Hospital Millennium Medical College with a history of visual loss of 7 years and bilateral lower limb weakness of 4 days duration. She had bilateral oculomotor nerve palsy. Her past medical history showed systemic hypertension for 18 years and dyslipidemia for 1 year. The objective evaluation of the patient revealed right optic nerve atrophy suggesting optic neuritis and flaccid paraplegia with sensory level at the fourth thoracic vertebra. Diagnostic work-up using electromyography and spinal magnetic resonance imaging revealed demyelinating anterior visual pathway dysfunction and signs of extensive cervicothoracic transverse myelitis from the third cervical to lower thoracic vertebrae, respectively. Then a diagnosis of neuromyelitis optica was established. After treatment with high-dose systemic steroid followed by azathioprine, the patient was stable for several months with significant improvement of vision and lower-extremity weakness with no relapse of symptoms.

**Conclusion:**

The case described here is a rare inflammatory demyelinating disorder of the central nervous system occurring in East Africa. It reminds clinicians to suspect neuromyelitis optica in a patient who presented with unexplained recurrent optic neuritis to make a timely diagnosis and prevention of permanent neuronal damage. Neuromyelitis optica can also be associated with oculomotor nerve involvement.

## Introduction

Neuromyelitis optica (NMO) or Devic’s syndrome is an autoimmune disease of the central nervous system (CNS) predominantly affecting the optic nerve and spinal cord [1]. The diagnosis of NMO requires the presence of optic neuritis, myelitis, spinal cord lesions involving three or more segments by magnetic resonance imaging (MRI), brain MRI not meeting the criteria of multiple sclerosis, and/or seropositive aquaporin-4 antibodies (AQP4-Abs) [2]. The discovery of NMO immunoglobulin G (IgG), directed against aquaporin-4 (AQP4), has changed the clinical definition of NMO and is important in the diagnostic criteria of the disease [1, 2]. However, in resource-limited settings, AQP4-Ab test is lacking and the diagnosis solely relies on the other clinical criteria. As far as the current literature, this is the third case of NMO reported from Ethiopia. It is also rarely reported from Sub-Saharan Africa. The low index of suspicion of such disease in our setup, together with diagnostic challenges, often leads to delay or misdiagnosis and worse clinical outcomes in patients that could have benefited from early treatment. We believe that the insights presented here might be useful to many other resource-limited settings.

## Case presentation

### Patient information

A 47-year-old woman from Addis Ababa presented to Saint Paul’s Hospital Millennium Medical College (SPHMMC) medical outpatient clinic in January 2020 with a compliant of bilateral lower limb weakness of 4 days duration. She gave history of significant reduction of vision in both of her eyes before 7 years for which she visited an ophthalmologist. At that time, optic neuritis was considered, and she was given systemic prednisolone for about 2 months after which her vision improved, but the patient was lost from follow-up. Four months ago, she developed complete loss of vision of both eyes for which she visited SPHMMC ophthalmology department when recurrent optic neuritis was again considered and restarted on systemic prednisolone. With subsequent follow-ups, her vision and oculomotor palsy in her left eye improved after 2 months of prednisolone therapy. Seven weeks later, she experienced right lower limb weakness followed by left lower limb weakness within 1 day and became bedridden. She also experienced radicular back pain, urinary retention, and constipation. She had no upper limb paresthesia or weakness. She had history of hypertension of 18 years and dyslipidemia of 1-year duration. For these, she was taking amlodipine, hydrochlorothiazide, alpha-methyldopa, and atorvastatin regularly. She had no history of joint pain or skin rash. She had no history of gastrointestinal surgery or chronic diarrhea. She reported no history of recent vaccinations or diabetes mellitus. She used to work as a janitor for a wine factory until 7 years ago when she lost her job because of her vision. She has two children (one male and one female).

### Clinical finding

She was conscious and alert. Her ophthalmic evaluation revealed vision of no light perception in both of her eyes. Intraocular pressure was 16 and 14 mmHg in her right and left eye, respectively. Slit lamp examination showed afferent pupillary defect and optic nerve atrophy in the right eye and sluggishly reactive pupil but relatively normal optic nerve appearance on the left eye. She had also bilateral oculomotor nerve palsy. Several weeks later, additional systemic findings were observed including bilateral lower limb weakness with muscle power of grade 0 on the Medical Research Council (MRC) scale, with depressed reflexes at the knees and ankles. In addition, the plantar reflexes were equivocal bilaterally. She had symmetrical sensory deficit below T4 to fine touch and pressure. Coordination test was normal.

### Timeline

**Table:** 

Case—previous history	A 47-year-old female patient who was relatively healthy 7 years back presented with reduced vision on both eyes that progressed to complete vision loss in her right eye. Her left eye vision showed some improvement following steroid therapy
Recent complaint (2/3/19–4/6/19)	Currently presented with 4 days history of bilateral lower extremity weakness Initially had paresthesia of lower extremities later lost sensation below her nipple line Inability to urinate and pass stool of the same duration
Physical examination	Symmetrical muscle bulk, no fasciculation Power 0/5 in bilateral lower extremity and 5/5 in both upper extremities Tone is hypotonic in both lower extremities and normotonic in both upper extremities Reflex is 1/4 in both ankle and knee, bilaterally sensory level is T4 Finger-to-nose test—normal Rapid alternating movement—intact
Investigation	CBC-WBC—11500 (n78, l17) HGB = 16.8 g/49.8, 16.2/39.4 MCV = 86.8, MCH = 29.3 PLT = 315,000 SGOT = 33, SGPT = 26, ALP = 117, T-BIL = 0.6, D-BIL = 0.2 Cr = 0.6, BUN = 23, Na = 130, K = 3.6, CL = 108 Hepatic viral markers negative ESR = 25 ANA negative, VDRL negative, RF negative CSF analysis—no cell, glucose = 82.4, protein = 24.8
Imaging	Brain MRI (taken 7 years back) normal, brain MRI (taken 4 months back) Few punctate T2/FLAIR hyperintense lesion within deep white matter of frontal and parietal lobe. Left temporoparietal volume loss Recent spinal MRI: expansile long segment T1 hypointense, T2 and STIR heterogeneously hyperintense lesion extending from C3 to lower thoracic vertebra No contrast enhancement

*Hx* History, *CBC* complete blood count, *WBC* white blood cell, **HGB** hemoglobin, *MCV* mean corpuscular volume, *MCH* mean corpuscular hemoglobin, *PLT* platelet, *SGOT* serum glutamic oxaloacetic transaminase, *SGPT* serum glutamic pyruvic transaminase, *ALP* alkaline phosphatase, *T-BIL* total bilirubin, *D-BIL* direct bilirubin, *Cr* creatinine, *BUN* Blood urea nitrogen, *Na* sodium, *K* potassium, *cl* chloride, *ESR* erythrocyte sedimentation rate, *ANA* antinuclear antibody, *VDRL* venereal disease research laboratory, *RF* rheumatoid factor, *STIR* short tau inversion recovery, *CSF* cerebrospinal fluid, *MRI* Magnetic resonance imaging, *FLAIR* fluid-attenuated inversion recovery

### Diagnostic assessment

**Fig. 1:**
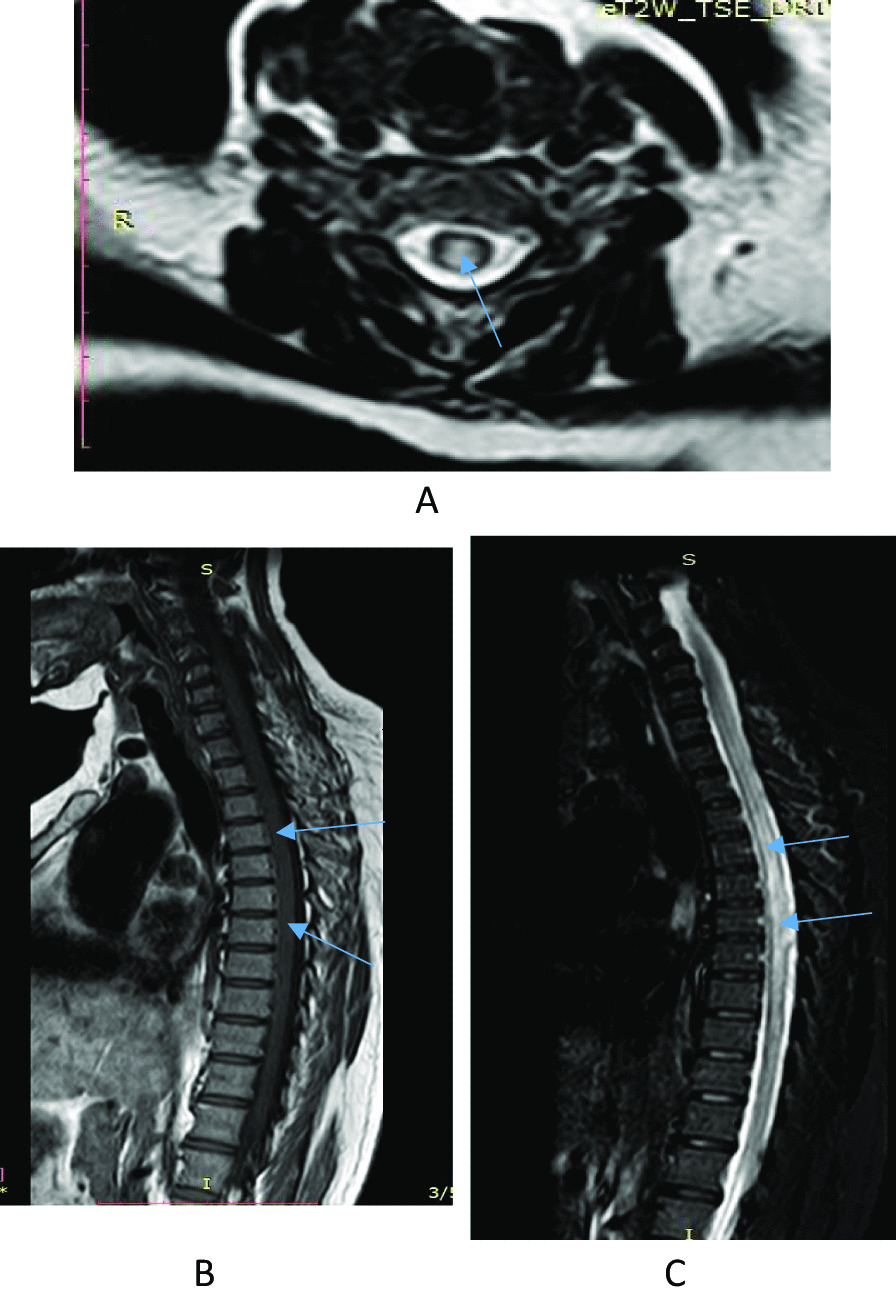
**A** Magnetic resonance imaging studies of axial T2W image showing hyperintense lesion over the spinal cord. **B** and **C** Shows Magnetic resonance imaging studies of Sagittal section showing expansible long segment T1 hypointense, T2 and STIR heterogeneously hyperintense lesion respectively extending from C3 to T12

**Fig. 2:**
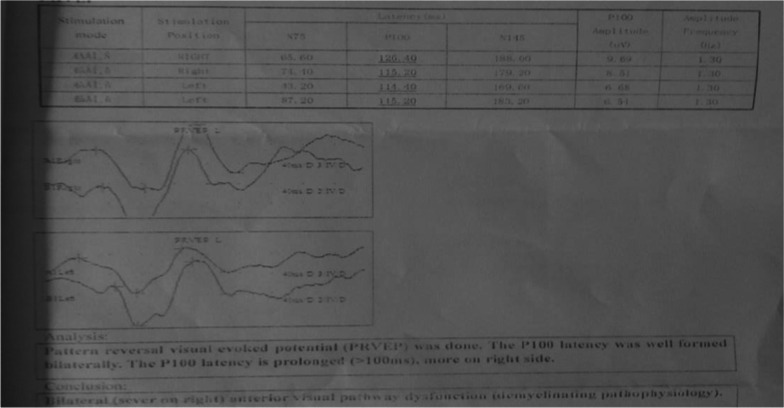
Electrophysiology: pattern reversal visual evoked potential (PRVEP) revealing prolonged P100 latency (> 100 ms), more on the right side

Her blood work-up showed normal complete blood count, liver and renal function tests, fasting blood sugar, and serum electrolytes. Echocardiography showed mild concentric left ventricular hypertrophy. Serologic tests for human immunodeficiency virus (HIV) and syphilis were negative. Cerebrospinal fluid (CSF) analysis showed clear and colorless appearance, and normal blood glucose level (82.4 mg/dl). However, the CSF protein content was elevated at 24.8 mg/dl, while the WBC count in the CSF was less than 5 cells/µl. Gram staining on her CSF was negative. The MRI of her cervicothoracic spine revealed expansible long segment T1 hypointense, T2 and short T1 inversion recovery (STIR) heterogeneously hyperintense lesion extending from C3 to T12 with no contrast enhancement (Fig. 1), suggesting probable myelitis or demyelination. There was no cord compressive lesion. Her brain MRI showed prominent sulci and cortical volume loss at the left parietotemporal lobe, and few punctuate T2/fluid-attenuated inversion recovery (FLAIR) hyperintense lesions within the deep white matter of the frontal and parietal lobes suggestive of nonspecific white matter lesion. Pattern reversal visual evoked potential (PRVEP) with electromyography revealed prolonged bilateral P100 latency (> 100 ms), more on the right side, suggesting bilateral (severe on the right side) anterior visual pathway dysfunction of demyelinating pathophysiology (Fig. 2). As a result, a diagnosis of neuromyelitis optica was made based on the diagnostic criteria without the NMO-IgG-antibody assays [2].

### Therapeutic intervention

The patient was started on intravenous methylprednisolone 1 g per day for 5 days followed by prednisolone 60 mg daily and subsequently discharged with azathioprine 75 mg daily. On discharge, she had left eye vision improvement.

### Follow up and outcome

On her follow-up, prednisolone was tapered and patient was discharged on azathioprine 75 mg daily. One year after discharge from the hospital, prednisolone was tapered to 7.5 mg daily and azathioprine reduced to 50 mg daily. Her vision in the right eye does not have light perception, but her left eye was 6/24. She showed complete recovery from oculomotor nerve palsy on her left eye. The lower limb weakness improved with power 3/5 on MRC scale. Additionally, there was resolution of urinary retention and sensory loss. Her recent visual evoked potential showed complete improvement of her left eye vision when compared with her previous visual evoked potential; however, her right anterior visual pathway dysfunction is still present.

## Discussion

Neuromyelitis optica is a severe immune-mediated inflammatory demyelinating disease of unknown etiology that predominantly affects the optic nerves and spinal cord [1]. The incidence of NMO in several studies ranged from 0.053 to 0.40 per 100,000 population, while the prevalence ranged from 0.52 to 4.4 per 100,000 population. The incidence is ten times higher in women than in men. Additionally, it is higher among Africans, Asians, and Latin Americans but is also common in Caucasians [2].

NMO is different from the classic relapsing-remitting multiple sclerosis (MS) with respect to pathogenesis, biomarkers, imaging features, and response to treatment. The target of the NMO antibody is aquaporin-4 (AQP4), a transmembrane protein that facilitates water transport in the CNS. AQP4 is highly expressed in the optic nerves, hypothalamus, brainstem, periventricular white matter, and gray matter of the spinal cord. However, only 70% of patients with NMO have positive AQP4 antibody. The remaining 30% do not express the antibody [3]. In our patient, AQP4 antibody test was not performed because it was lacking in the country at the time of diagnosis.

The main clinical features of the disorder are optic neuritis and extensive transverse myelitis. These two clinical events can occur simultaneously or separated by many years. Patients with optic neuritis can present with unilateral or bilateral eye pain and decreased vision that can rapidly progress to complete visual loss. It can occur before or after an attack of myelitis. On the other hand, patients with an attack of transverse myelitis may present with radicular back pain, symmetrical paraparesis or quadriparesis, bladder dysfunction, and sensory loss below the level of the spinal cord lesion. Less commonly, patients with brain involvement may experience intractable vomiting, hiccups, excessive daytime somnolence, reversible posterior leukoencephalopathy syndrome, neuroendocrine disorders, and (in children) seizures. One-third to half of patients with NMO may develop associated other autoimmune diseases such as systemic lupus erythematosus, Sjögren’s syndrome, autoimmune thyroiditis, myasthenia gravis, pernicious anemia, immune thrombocytopenic purpura, primary sclerosing cholangitis, and ulcerative colitis. More than 90% of patients with NMO have also recurrent symptoms [1–3]. The diagnosis of NMO requires the presence of two absolute criteria, plus at least two or three supportive criteria. The absolute criteria are optic neuritis and acute myelitis. The supportive criteria are: brain MRI finding not meeting criteria for MS at disease onset, spinal cord MRI with contiguous T2-weighted signal abnormality extending over three or more vertebral segments indicating a relatively large lesion in the spinal cord, and a positive aquaporin-4 antibody test. A case that fulfills the above criteria is 99% sensitive and 90% specific for the diagnosis of neuromyelitis optica [2]. CSF white cell counts (WBC) can be normal or mildly elevated in NMO (median 19 cells/μl, and > 100 cells/μl are possible during relapse in 35% of cases). An elevated CSF protein was observed in 50% of cases, and cytology often reveals lymphocytic, monocytic, neutrophilic, and eosinophilic cell types that are usually absent in MS. T2-weighted MRI that primarily involves the central gray matter of the spinal cord on axial sections is highly suggestive of NMO. NMO involves a longitudinally extensive cord lesion, often extending over three or more spinal cord segments. However, MS involves fewer than two spinal segments. The cervical cord is affected in 60% of cases, and lesions may extend into the medulla. At presentation, MRI of the brain may be normal in 55–84%, and brain involvement may occur in up to 85% of patients’ overtime [2, 4].

Our patient presented with recurrent bilateral optic neuritis of several years before the onset of transverse myelitis. The diagnosis of NMO was made based on the clinical findings of recurrent optic neuritis and first occurrence of transverse myelitis involving multiple segments (C3 to T12) of spinal cord on MRI. The brain MRI findings were also in favor of NMO than MS. Consistent with previous reports [1, 2], the CSF analysis also showed low white cell count and high protein content. In our patient, biomarkers like NMO-IgG, antinuclear antibody, anti-Smith antibody, and CSF oligoclonal bands could not be assessed for they were lacking in the country. In a previous report of NMO from Ethiopia, a 24-year-old woman from Southwest Ethiopia presented to a public hospital with progressive flaccid quadriparesis of 2 weeks and left optic neuritis of 1-week duration [5]. The other case report described a 27-year-old female with recurrent visual loss and progressive quadriplegia with suggestive MRI and visual evoked potential findings and positive aquaporin-4 antibody. In none of these reports was brainstem or oculomotor involvement reported [6].

Brainstem symptoms have been reported recently in NMO. A multicenter study involving 258 patients with NMO showed that brainstem signs were observed in 31.4% of the patients, with vomiting (33.1%) being the most common sign followed by hiccups (22.3%), oculomotor dysfunction (19.8%), pruritus (12.4%), hearing loss (2.5%), facial palsy (2.5%), vertigo or vestibular ataxia (1.7%), trigeminal neuralgia (2.5%), and other cranial nerve signs (3.3%). Most of these symptoms were reversible, but oculomotor dysfunction was persistent [7].

Another retrospective case study that evaluated 50 NMO patients showed 30% had brainstem involvement. The patients also had variable manifestations like in the previous study, including oculomotor dysfunction. The patient with oculomotor symptoms had lesions in the right cerebellar hemisphere and in the cerebral peduncle of the midbrain on brain MRI [8].

One of the most recent case reports published in 2019 showed a 61-year-old female who presented with an initial complaint of bilateral ptosis and diplopia without any motor or sensory symptoms. MRI showed mild lesions in the midbrain and third ventricle [9].

The main goals of treatment of NMO are to reverse symptoms and prevent future relapses. The treatment for acute attacks in a seropositive NMO is intravenous pulse methylprednisolone followed by oral prednisolone therapy, which will be tapered over several months after symptom resolution to prevent recurrence and complications. Patients who responded poorly to corticosteroid therapy are treated with intravenous immunoglobulin or plasmapheresis. NMO is associated with high relapse rate requiring maintenance immunosuppressive therapy with azathioprine and/or low-dose prednisolone. The treatment for seronegative NMO is similar to that of seropositive patients. In recent studies, rituximab was found to be an effective therapy for resistant or seronegative NMO patients. Patients who have frequent relapses during the first 2 years of follow-up, are severely sick at the initial attack, have late presentation, and have coexisting systemic lupus erythematosus or other autoimmune disorders were associated with poor prognosis [10].

## Conclusion

It is prudent to suspect NMO in a patient with unexplained recurrent optic neuritis. NMO should be considered as a cause for bilateral oculomotor palsy. Early initiation of further diagnostic work-up and aggressive immunosuppressive treatment are essential to prevent recurrences and permanent neurologic disability. It is very important to follow patients with optic neuritis for a longer period.

## Data Availability

Not applicable.

## Abbreviations

AQP4-Abs: Aquaporin-4 antibodies
AQP-4: Aquaporin-4
CSF: Cerebrospinal fluid
CNS: Central nervous system
FLAIR: Fluid-attenuated inversion recovery
HIV: Human immunodeficiency virus
IgG: Immunoglobulin G
MD: Medical doctor
MRI: Magnetic resonance imaging
MS: Multiple sclerosis
MRC: Medical Research Council
NMO: Neuromyelitis optica
PRVEP: Pattern reversal visual evoked potential
STIR: Short T1 inversion recovery
WBC: White blood cell count

## References

[1] Wingerchuk DM, Lennon VA, Lucchinetti CF, Pittock SJ, Weinshenker BG. The spectrum of neuromyelitis optica. Lancet Neurol. 2007;6:805–15. 10.1016/S1474-4422(07)70216-8 17706564

[2] Etemadifar M, Nasr Z, Khalili B, Taherioun M, Vosoughi R. Epidemiology of neuromyelitis optica in the world: a systematic review and meta-analysis. Mult Scler Int. 2015;2015:174720. 10.1155/2015/174720 PMC4417948 25973275

[3] Juryńczyk M, Craner M, Palace J. Overlapping CNS inflammatory diseases: differentiating features of NMO and MS. J Neurol Neurosurg Psychiatry. 2015;86:20–5. 10.1136/jnnp-2014-308984 25248365

[4] Wingerchuk DM, Banwell B, Bennett JL, Cabre P, Carroll W, Chitnis T, *et al*. International consensus diagnostic criteria for neuromyelitis optica spectrum disorders. Neurology. 2015;85:177–89. 10.1212/WNL.0000000000001729 PMC4515040 26092914

[5] Jemal A, Bane A, Ali S. A 24-year-old female with neuromyelitis optica from Ethiopia. Ethiop Med J. 2017;55(4):320–3.

[6] Heaton K, Shabana M, Gebreyohanns M. Diagnosis and treatment of neuromyelitis optica in Ethiopia. 2019;92(15 Supplement) P2.6-042.

[7] Kremer L, Mealy M, Jacob A, Nakashima I, Cabre P, Bigi S, Paul F, Jarius S, Aktas O, Elsone L, Mutch K, Levy M, Takai Y, Collongues N, Banwell B, Fujihara K, de Seze J. Brainstem manifestations in neuromyelitis optica: a multicenter study of 258 patients. Mult Scler. 2014;20(7):843–7. 10.1177/1352458513507822 24099751

[8] Jarius S, Kleiter I, Ruprecht K, Asgari N, Pitarokoili K, Borisow N, Hümmert MW, Trebst C, Pache F, Winkelmann A, Beume LA, Ringelstein M, Stich O, Aktas O, Korporal-Kuhnke M, Schwarz A, Lukas C, Haas J, Fechner K, Buttmann M, Bellmann-Strobl J, Zimmermann H, Brandt AU, Franciotta D, Schanda K, Paul F, Reindl M, Wildemann B, in cooperation with the Neuromyelitis Optica Study Group (NEMOS). MOG-IgG in NMO and related disorders: a multicenter study of 50 patients. Part 3: brainstem involvement—frequency, presentation and outcome. J Neuroinflamm. 2016;13(1):281. 10.1186/s12974-016-0719-z PMC5088671 27802825

[9] Yasuda K, Maki T, Takata M, Kimura K, Takahashi T, Kanbayashi T, Murase N, Ohtani R, Takahashi R, Nakamura M. Bilateral oculomotor nerve palsy in a case of anti-aquaporin-4 antibody-positive neuromyelitis optica spectrum disorder. J Clin Neurosci. 2019;66:271–2. 10.1016/j.jocn.2019.05.043 31178306

[10] Zéphir H, Bernard-Valnet R, Lebrun C, Outteryck O, Audoin B, Pittion S, Wierltewski S, Ouallet JC, Neau JP, Ciron J, Clavelou P, Mariginer R, Brassat D. Rituximab as first-line therapy in neuromyelitis optica: efficiency and tolerability. J Neurol. 2015;262:2329–35. 10.1007/s00415-015-7852-y 26194198

